# Does citation matter? Research citation in policy documents as an indicator of research impact – an Australian obesity policy case-study

**DOI:** 10.1186/s12961-018-0326-9

**Published:** 2018-06-28

**Authors:** Robyn Newson, Lucie Rychetnik, Lesley King, Andrew Milat, Adrian Bauman

**Affiliations:** 1 Sydney School of Public Health, Charles Perkins Centre D17, Level 6 Hub, The University of Sydney, Sydney, NSW 2006 Australia; 2 School of Medicine Sydney, University of Notre Dame Australia, 160 Oxford St, Darlinghurst, Australia

**Keywords:** Research impact, Policy, Public health research, Scientometrics, Bibliometrics

## Abstract

**Background:**

Citation of research in policy documents has been suggested as an indicator of the potential longer-term impacts of research. We investigated the use of research citations in childhood obesity prevention policy documents from New South Wales (NSW), Australia, considering the feasibility and value of using research citation as a proxy measure of research impact.

**Methods:**

We examined childhood obesity policy documents produced between 2000 and 2015, extracting childhood obesity-related references and coding these according to reference type, geographical origin and type of research. A content analysis of the policy documents examined where and how research was cited in the documents and the context of citation for individual research publications.

**Results:**

Over a quarter (28%) of the policy documents (*n* = 86) were not publicly available, almost two-thirds (63%) contained references, half (47%) cited obesity-related research and over a third (41%) of those containing references used unorthodox referencing styles, making reference extraction laborious. No patterns, in terms of the types of documents more likely to cite research, were observed and the number of obesity research publications cited per document was highly variable. In total, 263 peer-reviewed and 94 non-peer-reviewed obesity research publications were cited. Research was most commonly cited to support a policy argument or choice of solution. However, it was not always possible to determine how or why individual publications were cited or whether the cited research itself had influenced the policy process. Content analysis identified circumstances where research was mentioned or considered, but not directly cited.

**Conclusions:**

Citation of research in policy documents in this case did not always provide evidence that the cited research had influenced the policy process, only that it was accessible and relevant to the content of the policy document. Research citation across these public health policy documents varied greatly and is unlikely to be an accurate reflection of actual research use by the policy agencies involved. The links between citation and impact may be more easily drawn in specific policy areas or types of documents (e.g. clinical guidelines), where research appraisal feeds directly into policy recommendations.

## Background

Measuring the wider policy and practice impacts of health research is becoming an important field of study as researchers and funding bodies are increasingly required to demonstrate value for money, both in terms of generating new knowledge and contributing to health and economic outcomes [1]. The research impact assessment literature calls for metrics as proxy measures for the impacts of research, and the number of research citations in public policy documents has been suggested as one such indicator [2–4]. Presumably, if research is cited in a policy document, that research can be thought of as having an “*effect on, change or benefit to the policy*” and therefore an impact beyond the academic setting [5]. Reviewing sources cited in policy documents potentially offers a quantifiable, objective and simple method of measuring the impacts of research. The method has been proposed as less subjective and burdensome than relying on researchers themselves to recall and track the impacts of their research, particularly if the tracking of citation can be automated [6].

Only a few studies have examined the feasibility of extracting references from health policy documents [6–10]. These studies suggest that it is possible to use bibliometric techniques to identify the geographical origins of research, categorise the type of research cited (e.g. basic versus clinical research), and identify the funding sources of cited research. Quantifying citations of specific research outputs in a sample of policy documents has been incorporated into comprehensive research impact assessments [11–14]. To date, research citation studies have examined a limited range of health policy document types and areas of research, including clinical guidelines, on a range of topics [7–11, 13] and policy documents relevant to nursing home visiting research [14], drug monitoring systems [12], environmental health research [6] and climate change research [15].

Recent studies have investigated the feasibility of using automated approaches to policy document analysis, such as ‘text mining’ and reference list data extraction, to provide information about research impacts [6, 15]. Such methods rely on policy documents to be electronically accessible and to cite research in a manner that allows data extraction. It is likely that some policy documents are more suited to this type of analysis than others. For example, clinical guidelines are often readily available online, informed by an explicit process of evidence appraisal, and cite evidence in the form of a bibliography or database of sources [14]. Other types of policy documents, for example, government plans and strategies, may not share these characteristics. To interpret the findings of automated research citation assessment processes, it is important to understand their strengths and limitations with respect to different types of policy documents. This is an important area of research given that commercial applications of such technology, such as Altmetric, are already available and being utilised by researchers, universities and research funding bodies to track research impacts [16].

Existing studies suggest it is especially important to understand the research and policy context of research citation in policy documents [13, 15]. The citation of research in policy documents may be the result of different factors, including efforts by researchers to interact with policy-makers to disseminate their findings, or policy organisation-driven initiatives to include research in policy documents [15]. One hopes that research cited in policy documents was read, understood and considered in relation to the content of the policy. However, it can be difficult to determine the actual role played by cited research [14]. For example, research may be used in many ways by policy-makers, including instrumental uses, where research is explicitly or directly applied to address a policy problem; conceptual uses, where research has an influence on awareness, understanding or attitudes/perceptions amongst policy-makers generating ideas, arguments and criticisms which feed into policy debate; or symbolic uses, where research is used to justify a position or specific action already taken for other reasons or to attain specific goals based on a predetermined position [17]. In addition, many factors other than research contribute to policy development, including political ideology, stakeholder interests, community expectations and other competing information [14, 18].

In this paper, we investigate the feasibility of compiling and assessing research citations in a sample of public health policy documents and consider the utility of this approach as an indicator of research impact in the context of childhood obesity prevention policy in New South Wales (NSW), Australia, between 2000 and 2015. During this period, childhood obesity was a NSW Government public health priority (Box 1) and a wide variety of policy documents were produced. We examined the feasibility of retrospectively obtaining these policy documents, how these were accessed (publicly and by request), whether and how they cited research, the rates of citation, and the type of research cited. We also explored the document context of research citations to consider what this could reveal about how the research had been used.

## Methods

In considering policy documents as a source of information about research impact in the case of childhood obesity policy in NSW, we sought to answer the following research questions:

1. What types of public health policy documents were available, to what extent did these cite research, and how feasible was it to extract citation information from these documents?
2. What were the rates of research citation in these documents, and what type of research was cited?
3. Where and how was research cited, and what, in this case, did the citation of research indicate about research impacts?

### Policy document search

We searched for childhood obesity policy documents produced between 2000 and 2015 by NSW government agencies. Policy documents were defined as government documents in the form of a report, discussion paper, draft or final policy, formal directive, programme plan, strategic plan, budget bid, service agreement, implementation plan, guideline, or protocol with a focus on health service or programme design, delivery, evaluation or resourcing [19]. We excluded documents that did not mention obesity or were not relevant to children and young people (i.e. specifically targeted at another population group). We began with key policy documents known to the authors, and used a snowball technique to identify new documents mentioned in those already collated. This initial search was supplemented by further searches of current and archived NSW Government websites. Access to archived websites was provided by the NSW Health Library. Two current and two former public health employees of the NSW Ministry of Health reviewed the list of identified documents and made additional suggestions that led to further online searches. Documents that could not be located through internet searches, or via the NSW Health Library, were requested directly from the NSW Ministry of Health. Our search did not seek ministerial and cabinet briefing notes or internal meeting minutes as these documents were likely to be subject to Cabinet-in-confidence legislation.

### Policy document coding and reference extraction

A structured coding instrument was used to collate descriptive information about each policy document, including policy document title, date, author, type of document, availability, length, inclusion of any references, inclusion of childhood obesity references, and referencing style (e.g. reference list, footnote, hyperlinks or combination of these). The text of each document was scanned for passages or sections relevant to childhood obesity. References referring to childhood obesity or linked to statements relevant to childhood obesity, cited in these passages/sections, were identified. Details for these references were located through Scopus and exported or manually entered into a reference database. Extracted reference details included the publication title, date, authors’ names and affiliations, abstract, keywords, and publication source. Information on funding sources was rarely provided and was therefore not included in our analysis.

### Reference coding

A second coding instrument was developed to compile information about each reference, including type of publication/information source, geographical origin and type of research for research publications. We distinguished research from other referenced information sources (policies, media releases, information on websites, reference books) (see Table 1 for definitions). The research category was further subdivided into peer-reviewed research, non-peer-reviewed research and internal/external statistics. We further categorised peer-reviewed research according to whether the study was a single study, review/research synthesis or other. Finally, using an existing coding system, peer-reviewed research was categorised as descriptive research, intervention research, measurement research or other [20]. In most cases, the previously extracted reference details (which often included an abstract) provided enough information for coding. Where this was not the case, the primary source document was obtained. We also recorded, for each reference, the number of policy documents in which the publication had been cited.

**Table 1:** Definitions used for coding extracted research references

Geographical origin of publication	
International:	Document not Australian in origin. Research not conducted in Australia (for single studies) or none of the authors have Australian affiliations (for research synthesis and other)
Other Australian:	Document Australian in origin but not from a NSW source. Research conducted in Australia but not NSW (for singles studies) or any authors have Australian affiliations but not NSW affiliations (for research synthesis and other)
NSW:	Document from NSW source. Research conducted in NSW (for single studies) or any authors have NSW affiliations (for research synthesis and other)
Referenced information sources	
Research:	Publications reporting analyses of quantitative or qualitative data, development or application of theory [19], or discussions/commentaries about research and research methods. Including:
	*Internal/external statistics:* Statistical data from internal (e.g. NSW Health Survey) or external (e.g. Australian Bureau of Statistics) sources
	*Non-peer-reviewed research:* Research reported in technical monographs or in the grey literature
	*Peer-reviewed research:* Research reported in peer-reviewed journals
Policy documents:	Documents (report, discussion paper, draft or final policy, formal directive, programme plan, strategic plan, budget bid, service agreement, implementation plan, guideline or protocol with a focus on health service or programme design, delivery or resourcing [19]) authored by government agencies, non-government organisations or professional bodies where the purpose of the document was not to report research findings
Other information sources:	Cited information other than policy documents or research, e.g*.* media releases, information on websites, reference books, expert testimony
Peer-reviewed research	
Single study:	Publication reports the findings of a research study
Research synthesis:	Meta-analyses, systematic reviews, literature reviews
Other:	Commentaries, editorials, case reports, conference reports, conceptual models, health programme descriptions
Descriptive research:	Describes the nature, scope and correlates of the problem
Intervention research:	Evaluates the impact of an intervention, implementation under real world conditions, or the wide scale use of an intervention
Measurement studies:	Development of measures or testing the reliability, acceptability or validity of measurement instruments

### Policy document content analysis

For each policy document, we noted the structure and purpose of the document, the type of research that was used (if any), how different types of research were used and in what component of the document, and whether the processes for accessing any cited research had been described. In addition, we reviewed each citation occasion to note the way in which the research was cited (used to support a policy argument, decision or statement in the text), whether the research was cited alone or with other references, and whether the importance of the cited publication was explicitly stated. Illustrative examples of common citation contexts were identified and compiled.

## Results

### Availability of policy documents

**Fig. 1:**
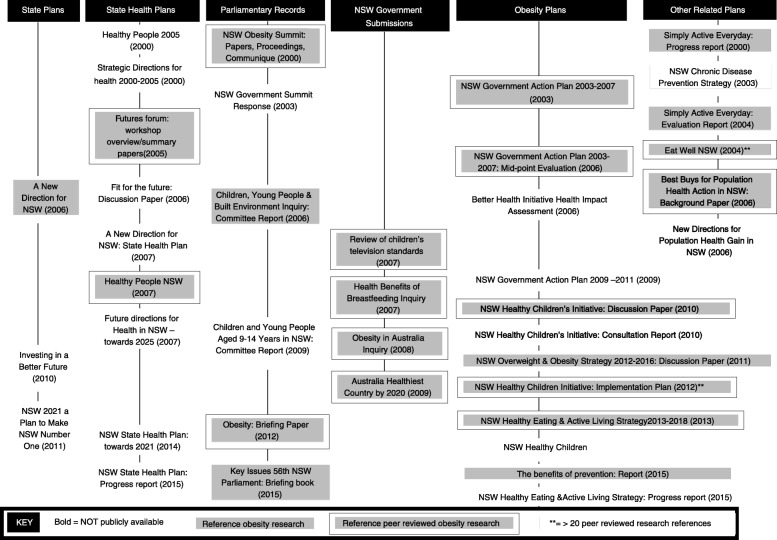
Policy documents by type, availability and whether research is cited – NSW government plans, strategies and parliamentary records

**Fig. 2:**
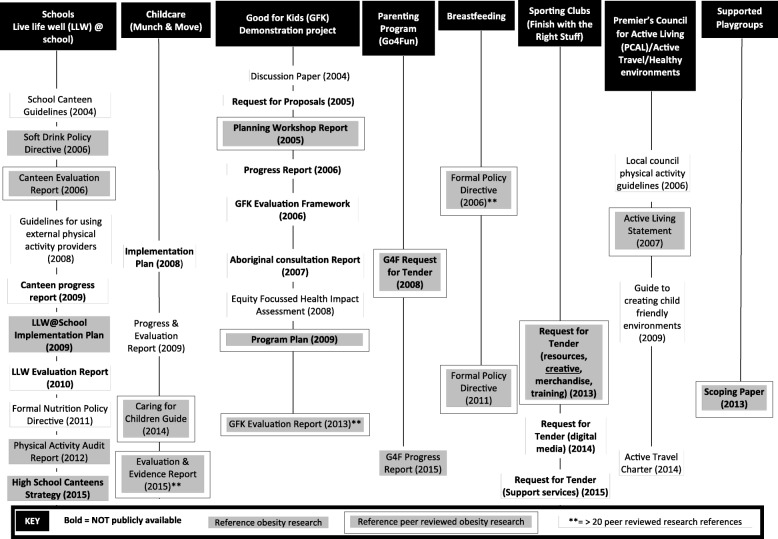
Policy documents by type, availability and whether research is cited – Specific initiatives

In total, 86 policy documents were obtained that included content about obesity applicable to children and young people. These documents related to NSW state plans (*n* = 3), NSW state health plans (*n* = 9), NSW parliamentary records (*n* = 11), NSW government submissions to Australian government processes (*n* = 5), NSW obesity plans and strategies (*n* = 12), other related plans and strategies (*n* = 6), and specific childhood obesity initiatives and programmes (*n* = 40). The documents included a range of document types such as inquiry, committee, consultation and evaluation reports, discussion papers, formal policy directives, programme and implementation plans, strategic plans, requests for tender and practice guides. An overview of the collated policy documents is provided in Fig. 1 (NSW government plans, strategies and parliamentary records) and Fig. 2 (Specific initiatives and programmes).

Nearly three-quarters (72%) of the policy documents were publicly available on existing and archived websites, while the remaining documents (28%) could not be found through public sources and were obtained on formal request from the NSW Ministry of Health (Table 2). Government plans, parliamentary records and submissions to parliamentary committees and inquiries, formal policy directives, guidelines and protocols could, in most cases, be obtained from public sources. Documents related to specific initiatives, such as reports of formative research, scoping papers, project plans, and progress and/or evaluation reports, were less often publicly available (Figs. 1 and 2).

**Table 2:** Summary of key findings for obesity policy documents obtained for analysis (*n* = 86)

	Yes		No	
	*n*	(%)	*n*	(%)
Document was publicly available	62	(72)	24	(28)
Document contained references	54	(63)	32	(37)
Document contained childhood obesity-related references (research and other information sources)	45	(52)	41	(48)
Document cited obesity-related research (peer-reviewed research, non-peer-reviewed research, internal/external statistics)	40	(47)	46	(53)
Document cited peer reviewed obesity research	27	(31)	59	(69)
Document cited non-peer reviewed obesity research	36	(42)	50	(58)
Document cited peer reviewed obesity research AND was publicly available	21	(24)	65	(76)

### Inclusion of references in policy documents

Nearly two-thirds of the identified documents (63%) contained references, half (47%) cited obesity-related research and less than a third (31%) cited peer-reviewed obesity research (Table 2). Over a third (*n* = 22/54; 41%) of the documents containing references did not list them in a single list or appendix, rather using footnotes, hyperlinks or a combination of methods. In these cases, reference extraction was time consuming.

There was no clear pattern in terms of the type of documents more likely to cite obesity research or peer-reviewed obesity research specifically (Figs. 1 and 2). Most documents citing non-peer-reviewed obesity research cited between one and ten such publications, while for peer-reviewed obesity research, there was more variation in the number of publications cited per document (Table 3). Only five documents referenced more than 20 peer-reviewed obesity research publications. These are highlighted in Figs. 1 and 2 and again cover a range of policy document types.

**Table 3:** Number of obesity research publications cited per document (*n* = 86)

	Number of documents										
	Range	0 refs		1–9 refs		10–19 refs		20–49 refs		50+ refs	
		*n*	%	*n*	%	*n*	%	*n*	%	*n*	%
Peer-reviewed research	0–57	59	(69)	15	(17)	7	(8)	4	(5)	1	(1)
Non-peer-reviewed research	0–11	50	(58)	33	(38)	3	(3)	–	–	–	–

### Type of references included in policy documents

In total, 526 unique references relevant to childhood obesity were extracted from the policy documents, of which half were peer-reviewed research publications (*n* = 263), close to a fifth were non-peer-reviewed research publications (*n* = 94), and 8% were internal/external data and statistics (*n* = 42). The remainder (*n* = 127) came from other information sources (e.g. policy documents, websites, books and media releases). Peer-reviewed research included journal articles and conference papers. Non-peer-reviewed research included population health status and survey reports (*n* = 39), programme evaluation and other research reports (*n* = 29), grey literature reviews (*n* = 22) and economic analyses (*n* = 4). While the total number of extracted peer-reviewed obesity research publications outnumbered the non-peer-reviewed publications by almost 3:1; the non-peer-reviewed research was referenced in a greater number of policy documents than peer-reviewed research (*n* = 36 compared to *n* = 27; Table 2).

Table 4 provides the geographical origin for the cited peer-reviewed and non-peer-reviewed obesity research. The non-peer-reviewed research was mostly Australian in origin, the NSW component of which comprised research almost exclusively commissioned or funded by the NSW Ministry of Health or local health districts. Overall, 40% of the cited peer-reviewed research was Australian in origin. The distribution of peer-reviewed research by type of study is provided in Table 5.

**Table 4:** Peer-reviewed and non-peer-reviewed obesity research by geographic origin of reference

Type of reference	International		Australian (excl NSW)		NSW		Total	
	*n*	%	*n*	%	*n*	%	*n*	%
Peer-reviewed research	159	(60)	54	(21)	50	(19)	263	100
Non-peer-reviewed research	13	(14)	38	(40)	43	(46)	94	100

**Table 5:** Peer-reviewed obesity research by type of study

Type of reference	Descriptive		Intervention		Other		Total	
	*n*	%	*n*	%	*n*	%	*n*	%
Single study	89	(58)	62	(40)	3	(2)	154	(59)
Research synthesis	31	(44)	40	(56)	–	–	71	(27)
Other peer review	–	–	–	–	38	(100)	38	(14)
Total	120	(46)	102	(39)	41	(15)	263	(100)

### Frequency of citation

Only 20% of the extracted peer-reviewed obesity research publications were cited in more than one policy document (Table 6). A list of the most frequently cited peer-reviewed publications is provided in Table 7. Most of these were descriptive research publications.

**Table 6:** Frequency of citation of specific obesity research publications across policy documents

Type of reference	Frequency of citation of each publication										
	One document		Two documents		Three documents		Four documents		Five+ documents		Total
	*n*	%	*n*	%	*n*	%	*n*	%	*n*	%	*n*
Peer reviewed publications	211	(80)	39	(15)	4	(2)	5	(2)	4	(2)	263
Non-peer reviewed publications	68	(72)	17	(18)	4	(4)	1	(1)	4	(4)	94

**Table 7:** Peer-reviewed research publications cited in four or more policy documents

Title	Date	Geographical origin	Number of citing docs
Risks and consequences of childhood and adolescent obesity [41]	1999	International	8
Childhood obesity: Public-health crisis, common sense cure [42]	2002	International	8
Prevalence of overweight and obesity in Australian children and adolescents: Reassessment of 1985 and 1995 data against new standard international definitions [43]	2001	Australian	5
Health consequences of obesity [44]	2003	International	5
Fat, friendless and unhealthy: 9-year old children’s perception of body shape stereotypes [45]	1995	International	4
Obesity prevention: The case for action [46]	2002	International	4
Change in the prevalence of overweight and obesity among young Australians, 1969–1997 [47]	2003	Australian	4
Does overweight in childhood have an impact on adult health? [48]	2003	International	4
Reducing obesity in early childhood: Results from Romp & Chomp, an Australian community-wide intervention programme [49]	2010	Australian	4

### Context of research citation

#### Citation uses of different types of research

Descriptive research was typically cited in relation to statements outlining the policy rationale. Such statements described the size of the problem, prevalence of risk factors, beliefs and attitudes around obesity, the causes and consequences of obesity and in some cases who should be targeted. It was common for this type of research to be cited in policy documents related to broader strategies and plans, as well as specific initiatives. No patterns in relation to the citation of single studies versus research syntheses in different types of policy documents were observed for descriptive research.

Intervention research was more commonly cited in relation to statements outlining the policy options, chosen solutions or to substantiate evaluation findings. Very few government plans and strategies cited single intervention studies, usually citing evidence reviews or broad overviews. Conversely, documents related to specific initiatives more commonly cited single intervention studies or reviews that focused on specific interventions or settings (e.g. interventions to promote physical activity in childcare or school-based interventions).

#### Requirements and criteria for considering research

The policy documents did not reveal any explicit requirements to consider research. However, it was apparent that the policy-makers involved in developing many of the policy documents had engaged in processes to consider research or to consult with researchers, for example, by convening stakeholder discussions and consultation or planning workshops that included researchers prior to the development of specific plans and strategies. Policy agencies also commissioned literature reviews, and parliamentary inquiries or committees were another mechanism through which researchers had input into the policy process. The fact that these processes had been explicitly described did not always influence the subsequent citation of the research that had been considered.

In addition, the policy documents rarely revealed whether specific criteria for evidence appraisal had been used (e.g. currency, quality, local relevance) although, in some instances, such criteria may have been explained elsewhere (e.g. in the terms of reference for commissioned reviews). One exception was identified:

“*The priority of interventions was identified with consideration of promise of effectiveness, feasibility and sustainability of implementation, acceptability to the community, and equality of outcomes across population groups. The top ten interventions in each setting …, based on cumulative scores against these criteria, represent portfolio priorities for the Area Health Service.*” (NSW Health. Hunter New England Child Obesity Prevention Program Portfolio Planning Workshop Report (2005), Unpublished).

#### Influence of specific publications

In most instances, research was cited to support a policy argument or decision. There were examples where it was clear that the cited research was used to directly inform the policy argument or choice of solution. For example, in relation to the Go4Fun programme, the authors of the Healthy Children’s Initiative Implementation Plan (2012) [21] explicitly stated that this programme was adapted from an existing evidence-based programme and cited research related to this. In other instances, it was difficult to say with certainty that the cited research itself had influenced the policy direction or awareness and knowledge of the issue, and only that it was used to support the argument being put forward in the policy document, for example, for the Go4Fun programme, the citation of a research study from 1988 in relation to parental involvement with children’s health promotion:

“*Many commercial providers offer face-to-face nutrition, physical activity or healthy weight advice but these services tend to primarily address the needs of adults. In contrast, Go4Fun*^*®*^ *aims to address the needs of overweight and obese children (7 to 13 years) and their parents/carers by assisting them to develop a long-lasting healthy approach to living. Research suggests that parental involvement is crucial for the implementation and maintenance of new health behaviours in younger children.*” (Healthy Children’s Initiative: NSW Implementation Plan (2012), [21])

In addition, some policy documents included tangential areas of content, where the research cited appeared to have limited relevance to the overall policy position; for example:

“*Commonly a BMI-for-age is calculated by the 85th percentile being the cut off point for overweight and above the 5th percentile being the cut off point for being underweight (Gill, King, & Webb, 2005).*” (NSW Government Submission to the Inquiry into Obesity in Australia (2008), [22])

Commissioned reviews appeared to have played an important role in informing policy development. There were examples where this role was explicitly stated within the policy document:

“*The Evidence Update on Obesity Prevention, completed by the Physical Activity and Nutrition Research Group PANORG, provides a summary of the evidence to guide the development of the NSW Overweight and Obesity Strategy 2012–2016. The evidence update draws upon a broad range of research studies and policy analyses, including current national and state-wide strategies, to inform policy and practice.*” (NSW Department of Health. NSW Overweight and Obesity Strategy 2012–2016: Discussion Paper (2011). Unpublished).

It may be hypothesised that the number of policy documents citing a research publication reflects the publication’s perceived importance and influence, as is likely to be the case, for example, for the NSW Schools Physical Activity and Nutrition Survey Reports [23, 24]. This research provided quality data on objectively measured NSW childhood obesity prevalence and was frequently cited and explicitly used to set targets, monitor progress and guide implementation approaches.

Research about the consequences of obesity and the links between childhood obesity and adult health problems was often used to highlight the need for action, and several publications addressing this topic were frequently cited (Table 7). However, compared to the NSW Schools Physical Activity and Nutrition Survey example above, it was not clear why these publications were cited so often in preference to other similar publications. There was no indication whether the frequent citation of these publications reflected the quality and prominence of the research. ‘Historical precedence’ may have played some role, that is, the research may have been repeatedly cited based on its use to support similar statements in previous policy documents.

#### Source of cited research

How the research came to be cited in a policy document may reveal some information about the influence of the research on the policy direction. However, in most instances, it was not possible to determine how the policy document authors had come to know about the research they cited. Exceptions were documents relating to parliamentary processes (e.g. parliamentary inquiry and committee proceedings and reports), where specific researchers or research groups were sometimes mentioned in verbal proceedings or via submissions, either discussing their own research or other research. It was possible to determine that a research publication was subsequently cited in a report because it had been presented to the committee in this way; for example:

“*The NSW Centre for Overweight and Obesity report, Creating Healthy Environments: a review of the links between the physical environment, physical activity and obesity, and the Centre’s submission to the inquiry stress the complex relationship between the physical environment, physical activity and childhood obesity*” (Children, Young People & Built Environment Inquiry: Committee Report (2006), [25])

There were also instances where it was clear that the research had been cited because it had been commissioned by the policy agency; for example:

“*In association with this work, the NSW Centre for Public Health Nutrition … (based at the University of Sydney and funded by NSW Health) has produced a series of reports to inform policy development. NSW Health acknowledges that these reports have provided the basis for much of the information in this submission and has been extracted verbatim where relevant.*” (Submission to National Breastfeeding Inquiry (2007), [26])

#### Research use not reflected in citation rates

Examining the text of the policy documents identified instances where research findings had been quoted or mentioned without referencing the source of that information; for example:

“*In 2004, almost a quarter of children and young people (5–16 years) were overweight or obese and at least half of NSW adults are outside the healthy weight range.*” (Healthy People NSW (2007), [27])

“*Randomised controlled trials conducted in NSW by academic consortia have demonstrated promising results in regards to reducing participating children's body mass index and waist circumference. The programs included activities for children and educational programs for parents.”*, NSW Health. Health Administration Corporation Tender No: DOH 08/27. Request for Tender: Development and delivery of NSW parenting program to address childhood overweight and obesity. Information package for tenderers. Unpublished).

There were also instances where research may have been utilised but not cited because it was included within other documents; for example, some policy documents cited reviews commissioned by the NSW Ministry of Health as well as key individual studies included in that review, whereas, in other examples, only the primary review was cited. Where only the primary review was cited, the influence of any individual studies utilised by policy-makers as a result of reading the review could not be determined. The citation of other policy documents which were themselves informed by research provides another example. The Health Promoting Schools Framework, Australian Dietary Guidelines for Children and Adolescents, and Physical Activity Recommendations for Children and Young People, and the World Health Organization Global Strategy on Diet, Physical Activity and Health, were frequently cited in policy documents to indicate that the strategies chosen were consistent with evidence-based recommendations or practice.

Finally, some policy documents referred to previous processes for considering evidence, rather than citing research directly, for example:

“*In the course of this state-wide whole-of-health-system planning project, significant research was conducted involving examination of local, interstate, national and international health policies, planning and research literature.*” (Future Directions for Health in NSW – towards 2025, (2007), [28])

## Discussion

Policy documents are a commonly used source of information in research impact assessments [29, 30]. Most assessments start with a specific research project and trace that forward to identify its impact [31]; for example, an Australian study utilised this forward tracing approach to assess the impacts of a population-wide series of obesity monitoring surveys [32]. In the current study, we used a ‘backward tracing’ approach to examine 15 years of NSW childhood obesity policy documents as the starting point for analysis to determine what information these documents could provide about the utilisation and impact of research.

We found that it was possible to locate a range of policy documents covering our study period. However, over a quarter were not publicly available, only two-thirds contained references, just under half cited obesity research and over a third of the documents containing references used unorthodox referencing styles. There did not appear to be any patterns in terms of the types of documents more likely to cite research, and the number of research citations in each document was highly variable. It was possible to collate reference information, although this was difficult and time consuming where references were not contained in a single list.

In total, 263 unique peer-reviewed and 94 non-peer-reviewed publications were cited, of which only a fifth of peer-reviewed and just over a quarter of non-peer-reviewed publications were cited in more than one policy document. The cited peer-reviewed research included almost equal numbers of descriptive research and intervention research publications. Descriptive research was used to support a policy rationale, while intervention research was used to support policy options, chosen solutions or to substantiate evaluation findings. Both single research studies and reviews of the literature were cited. Overall, 40% of the cited peer-reviewed research and twice that proportion of non-peer reviewed research was Australian in origin, possibly reflecting the importance of locally relevant research to the policy process or knowledge of local research amongst local policy-makers [8, 9]. Cited non-peer reviewed research commissioned by NSW policy agencies appeared to have been particularly influential in terms of informing the policy process. Research was not the only form of information cited in policy documents reflecting the many sources of information considered by policy-makers, including information from monitoring studies and population-based surveys, government reports, stakeholder consultation, other policy documents and expert testimony [18, 33].

Our findings raised several issues related to the limitations of this methodology as a measure of research impact, when used in isolation. Firstly, citation of a publication did not necessarily indicate that the research had influenced the policy process [14]. Sometimes, the role individual publications played in the policy process was explicitly stated, while in other cases it was only possible to say with certainty that the publication was cited to establish credibility for the argument being put forward in the policy document. Research was also sometimes used to support statements that were tangential and not relevant to the overall policy direction. This is not entirely dissimilar to the ways in which research may be used and cited in academic publications. In academic publications, some cited research will have provided a foundation for the research under study or contributed to the researcher’s knowledge of the topic, but some will have been cited for comparative purposes, or as contrasts. We would suggest that research citation in a policy document is an indication of the research publication’s accessibility to the document authors and its relevance to the policy topic, but not necessarily that the research had an impact on policy (defined as an “*effect on, change or benefit to the policy*” [5]). In addition, it was difficult to determine the nature of any effect. For example, distinguishing between whether the cited research had been utilised in a tactical or symbolic way to support a predetermined position, or whether it had directly contributed to the policy’s adoption and development (instrumental use) was very challenging. Certainly, determining whether the cited research had influenced the knowledge, ideas and awareness of issues amongst the policy-makers involved (conceptual use) was not possible utilising this method. Other evidence, such as the testimony of policy-makers, may be required to confirm whether the cited research had an influence on policy directions, how it was utilised and the extent of that influence.

A second issue is that the method identified research that was cited, but not necessarily all the research that was considered during policy development. Sometimes, research was mentioned without being cited, or accessed and considered through other documents (e.g. systematic reviews or other policy documents) or processes (e.g. consultations) but not cited directly. In addition, not all policy deliberations will be recorded in a formal way, so policy documents only provide a partial record of policy discussions, some types more so than others. For example, legislative documents may provide a more fulsome account of policy deliberations, including any information presented, as records of verbal proceedings and written submissions are kept [34–36]. Citation rates are likely to provide an underestimation of research use by policy agencies and the method has the potential to miss research that was in fact impactful, and place undue importance on cited research. It may be that the method is more suited to policy documents that utilise a more consistent approach to the consideration, appraisal and citation of research. Certainly, studies examining research cited in clinical guidelines do not leave as many questions unanswered, as our study has, regarding the base of research considered and the process for appraising and including research that is cited [7–9].

Finally, the high degree of variability in this sample of policy documents in terms accessibility and research citation patterns and styles has implications for automated citation extraction systems. Only some of the policy documents we examined would have been suitable for or yielded results from automated data extraction, thus producing rather hit and miss outcomes for identifying impactful research publications. Whether the variability we encountered would be found in other public health policy areas warrants further investigation. However, it is likely that there will be differences between policy areas which will influence citation rates and the utility of automated systems. This is an important issue if rates of citation of research in policy documents are ever to be compared across research disciplines (e.g. clinical compared to public health research).

### Limitations of this study

We have focused on a single policy issue in one Australian jurisdiction. The types and requirements of documents obtained for this study may differ from policy documents available for other public health policy areas or other jurisdictions. The findings of this case study are not intended to be generalised, but rather indicative. Within this case study, we attempted to obtain as many policy documents as possible, but some policy documents may have been missed. Recall of policy documents was difficult for some policy-makers we asked due to the timeframe of the study. Prospective collation of documents as they are produced would eliminate some of these issues. In addition, only one author completed the coding of policy documents and references, which may have introduced some bias, although strict coding definitions were developed and implemented.

## Conclusions

The analysis of citation of research in policy documents performed herein indicated that research had been accessed and considered, but further qualitative investigation is required to answer the more important questions of why and how the research was used. Rather than being used as a definitive indicator of impact, automated citation searches may be useful in terms of pointing researchers in the right direction, so that they can investigate what role their research played in a policy process. Nevertheless, the links between citation and impact may be more easily drawn in other policy areas (e.g. clinical guidelines), where research appraisal feeds more directly into policy recommendations.

While highlighting instances of use of specific research products, research citation across the public health policy documents included here varied greatly and is unlikely to be an accurate reflection of actual research use by the policy agencies involved. Research citation in policy documents in this instance said more about the policy document than research utilisation and this may also be the case for other public health policy areas.

## Box 1: Overview of the NSW childhood obesity policy context

The NSW State Government was the first in Australia to identify and respond to the need for a coordinated approach to obesity prevention. In 2002, the Childhood Obesity Summit brought together key stakeholders to develop an across-government, inter-sectoral response to childhood obesity, culminating in the development and implementation of Prevention of Obesity in Children and Young People: NSW Government Action Plan 2003–2007 [37]. This plan aimed to raise community awareness and developed the platform for a coordinated cross-agency commitment to the implementation of obesity prevention strategies. Subsequently, targets for reducing childhood obesity rates have been included in successive NSW Government State Plans, which set the strategic direction for all NSW government agencies and services, and integrated as priorities in NSW State Health Plans. An increase in national and state funding enabled the expansion of existing initiatives and the introduction of new programmes [21, 38]. Tackling Childhood Obesity is currently (since September 2015) one of the 12 Premier’s Priorities for improving outcomes for the people of NSW [39].

The NSW Ministry of Health has been the lead agency for delivery of obesity strategies in NSW, supported by governance structures to ensure coordination and accountability for actions across all NSW government agencies. Initiatives to address childhood obesity have been implemented utilising a settings-based approach with a focus on homes and communities, healthcare environment, schools, childcare and children’s services, and the urban environment. Programmes are run at State and local health district levels and include a mix of whole of population and targeted approaches to obesity prevention. Another identified priority area has been knowledge production and research infrastructure development. The NSW Government funds the Schools Physical and Activity and Nutrition Survey, to provide a mechanism for objectively monitoring overweight and obesity rates for children and young people in NSW, as well as the Physical Activity Nutrition and Obesity Research Group at the University of Sydney [40].

These investments in NSW have run parallel to national obesity strategies including successive national obesity plans and a parliamentary inquiry into obesity in 2008. National activities have focussed on community education, including social marketing campaigns and national physical activity and nutrition guidelines for children and young people. The Australian Government has responsibility for food taxation and marketing legislation; however, no changes in this area have been made to date.

## Abbreviation

NSW: New South Wales

## References

[1] Martin BR (2011). The research excellence framework and the ‘impact agenda’: are we creating a Frankenstein monster?. Res Evaluation.

[2] Buxton M (2011). The payback of ‘payback’: challenges in assessing research impact. Res Eval.

[3] Thonon F, Boulkedid R, Delory T, Rousseau S, Saghatchian M, Van Harten W (2015). Measuring the outcome of biomedical research: a systematic literature review. PLoS One.

[4] Sarli CC, Dubinsky EK, Holmes KL (2010). Beyond citation analysis: a model for assessment of research impact. J Med Library Assoc.

[5] Research Excellence Framework (2014). Assessment Framework and Guidance on Submissions..

[6] Drew CH, Pettibone KG, Finch FO, Giles D, Jordan P (2016). Automated research impact assessment: a new bibliometrics approach. Scientometrics.

[7] Kryl D, Allen L, Dolby K, Sherbon B, Viney I (2012). Tracking the impact of research on policy and practice: investigating the feasibility of using citations in clinical guidelines for research evaluation. BMJ Open.

[8] Lewison G, Sullivan R (2008). The impact of cancer research: how publications influence UK cancer clinical guidelines. Br J Cancer.

[9] Grant J, Cottrell R, Cluzeau F, Fawcett G (2000). Evaluating “payback” on biomedical research from papers cited in clinical guidelines: applied bibliometric study. BMJ.

[10] Lewison G (2003). Beyond outputs: new measures of biomedical research impact. Aslib proceedings.

[11] Bunn F, Trivedi D, Alderson P, Hamilton L, Martin A, Iliffe S (2014). The impact of Cochrane systematic reviews: a mixed method evaluation of outputs from Cochrane review groups supported by the UK National Institute for Health Research. Syst Rev.

[12] Ritter A, Lancaster K (2013). Measuring research influence on drug policy: a case example of two epidemiological monitoring systems. Int J Drug Policy.

[13] Hanney SR, Watt A, Jones TH, Metcalf L (2013). Conducting retrospective impact analysis to inform a medical research charity’s funding strategies: the case of Asthma UK. Allergy Asthma Clin Immunol.

[14] Bunn F, Kendall S (2011). Does nursing research impact on policy? A case study of health visiting research and UK health policy. J Res Nurs.

[15] Bornmann L, Haunschild R, Marx W (2016). Policy documents as sources for measuring societal impact: how often is climate change research mentioned in policy-related documents?. Scientometrics.

[16] www.altmetric.com. Accessed 20 Dec 2016.

[17] Lavis JN, Ross SE, Hurley JE (2002). Examining the role of health services research in public policymaking. Milbank Q.

[18] Bowen S, Zwi AB, Sainsbury P, Whitehead M (2009). Killer facts, politics and other influences: what evidence triggered early childhood intervention policies in Australia?. Evid Policy.

[19] Haynes A, Turner T, Redman S, Milat AJ, Moore G (2015). Developing definitions for a knowledge exchange intervention in health policy and program agencies: reflections on process and value. Int J Soc Res Methodol.

[20] Milat AJ, Bauman AE, Redman S, Curac N (2011). Public health research outputs from efficacy to dissemination: a bibliometric analysis. BMC Public Health.

[21] Implementation Plan for The Healthy Children Initiative National Partnership Agreement on Preventive Health Canberra: Australian Government; 2012. http://www.federalfinancialrelations.gov.au/content/npa/health/_archive/healthy_workers/healthy_children/NSW_IP_2013.pdf. Accessed 20 Dec 2016.

[22] NSW Government (2008). NSW Government Submission to the Parliament of Australia House of Representatives Standing Committee on Health and Ageing Inquiry into Obesity in Australia.

[23] Hardy LLKL, Espinel P, Cosgrove C, Bauman A (2011). NSW Schools Physical Activity and Nutrition Survey (SPANS) 2010: Full Report. Sydney: NSW Ministry of Health.

[24] Booth MOA, Denney-Wilson E, Hardy L, Yang B, Dobbins T (2006). NSW School Physical Activity and Nutrition Survey (SPANS) 2004: Full Report.

[25] New South Wales Parliament (2006). Committee on Children and Young People. Inquiry into Children, Young People and the Built Environment/Parliament of New South Wales, Committee on Children and Young People (report; no.8/53).

[26] NSW Health (2007). NSW Health Submission to the Australian Parliament House Standing Committee on Health and Ageing Inquiry on the Health Benfits of Breastfeeding (Submission 479).

[27] Population Health Division (2007). Healthy people NSW: Improving the health of the population.

[28] NSW Department of Health (2007). Future Directions for Health in NSW - towards 2025. Fit for the Future.

[29] Milat AJ, Bauman AE, Redman S (2015). A narrative review of research impact assessment models and methods. Health Res Policy Syst..

[30] Raftery J, Hanney S, Greenhalgh T, Glover M, Blatch-Jones A (2016). Models and applications for measuring the impact of health research: update of a systematic review for the health technology assessment programme. Health Technol Assess.

[31] Boaz A, Fitzpatrick S, Shaw B (2009). Assessing the impact of research on policy: a literature review. Sci Public Policy.

[32] Laws R, King L, Hardy LL, Milat A, Rissel C, Newson R, Rychetnik L, Bauman AE (2013). Utilization of a population health survey in policy and practice: a case study. Health Res Policy Syst.

[33] Zardo P, Collie A (2014). Measuring use of research evidence in public health policy: a policy content analysis. BMC Public Health.

[34] Kite HA, Gollust SE, Callanan RA, Weisman SR, Benning SJ, Nanney MS (2014). Uses of research evidence in the state legislative process to promote active environments in Minnesota. Am J Health Promot.

[35] Gollust SE, Kite HA, Benning SJ, Callanan RA, Weisman SR, Nanney MS (2014). Use of research evidence in state policymaking for childhood obesity prevention in Minnesota. Am J Public Health.

[36] Apollonio DE, Bero LA (2009). Evidence and argument in policymaking: development of workplace smoking legislation. BMC Public Health.

[37] NSW Department of Health. Prevention of Obesity in Children and Young People: NSW Government Action Plan 2003–2007. NSW Department of Health: Sydney 2003 http://web.archive.org/web/20080725091243/http://www.health.nsw.gov.au/obesity/adult/gap/ObesityActionPlan.pdf. Accessed 20 Dec 2016.

[38] The Department of Health. Australian Governenment. COAG Health Services - Promoting Good Health, Prevention and Early Intervention. http://www.health.gov.au/internet/budget/publishing.nsf/Content/budget2006-hfact37.htm. Accessed 20 Dec 2016.

[39] NSW Government. NSW Premier's Priorities. https://www.nsw.gov.au/improving-nsw/premiers-priorities/. Accessed 20 Dec 2016.

[40] University of Sydney. Physical Activity Nutrition and Obesity Research Group (PANORG). http://sydney.edu.au/medicine/public-health/prevention-research/research/panorg.php. Accessed 20 Dec 2016.

[41] Must A, Strauss RS (1999). Risks and consequences of childhood and adolescent obesity. Int J Obes Relat Metab Disord.

[42] Ebbeling CB, Pawlak DB, Ludwig DS (2002). Childhood obesity: public-health crisis, common sense cure. Lancet.

[43] Magarey AM, Daniels LA, Boulton TJ (2001). Prevalence of overweight and obesity in Australian children and adolescents: reassessment of 1985 and 1995 data against new standard international definitions. Med J Aust.

[44] Reilly JJ, Methven E, McDowell ZC, Hacking B, Alexander D, Stewart L (2003). Health consequences of obesity. Arch Dis Child.

[45] Hill AJ, Silver EK (1995). Fat, friendless and unhealthy: 9-year old children's perception of body shape stereotypes. Int J Obes Relat Metab Disord.

[46] Kumanyika S, Jeffery RW, Morabia A, Ritenbaugh C, Antipatis VJ (2002). Obesity prevention: the case for action. Int J Obes Relat Metab Disord.

[47] Booth ML, Chey T, Wake M, Norton K, Hesketh K, Dollman J (2003). Change in the prevalence of overweight and obesity among young Australians, 1969-1997. Am J Clin Nutr.

[48] Must A (2003). Does overweight in childhood have an impact on adult health?. Nutr Rev.

[49] de Silva-Sanigorski AM, Bell AC, Kremer P, Nichols M, Crellin M, Smith M (2010). Reducing obesity in early childhood: results from Romp & Chomp, an Australian community-wide intervention program. Am J Clin Nutr.

